# Enhanced Measurement of Sugar-Sweetened Beverage Marketing to Young Immigrant Children in Grocery Store Environments

**DOI:** 10.3390/nu15132972

**Published:** 2023-06-30

**Authors:** Hadis Dastgerdizad, Rachael D. Dombrowski, Noel Kulik, Kathryn A. G. Knoff, Bree Bode, James Mallare, Dariush K. Elyaderani, Ravneet Kaur

**Affiliations:** 1 Department of Public Health, University of South Carolina, Bluffton, SC 29909, USA; 2 College of Education, Health and Human Services, California State University-San Marcos, San Marcos, CA 92096, USA; rdombrowski@csusm.edu; 3 Center for Health and Community Impact, Division of Kinesiology, Health & Sport Studies, College of Education, Wayne State University, Detroit, MI 48202, USA; ab7564@wayne.edu; 4 Office of Policy Support, Food and Nutrition Service, US Department of Agriculture, Alexandria, VA 22314, USA; kathryn.knoff@usda.gov; 5 Michigan Fitness Foundation, Lansing, MI 48314, USA; bbode@michiganfitness.org; 6 Department of Family Medicine and Public Health Sciences, Wayne State University, Detroit, MI 48202, USA; jpmallare@wayne.edu; 7 Chapman School of Business, Florida International University, Miami, FL 33199, USA; 8 Division of Health Research and Evaluation, Department of Family and Community Medicine, College of Medicine, University of Illinois, Rockford, IL 61107, USA; ravneetk@uic.edu

**Keywords:** obesogenic environment, sugar-sweetened beverage marketing, early childhood obesity, immigrant enclaves, nutrition environment measures survey, independently owned grocery stores

## Abstract

The marketing of Sugar-Sweetened Beverages (SSBs) within grocers is an obesogenic factor that negatively impacts children’s nutritional behavior, specifically for people from racial and ethnic minority groups, such as immigrants. We aimed to develop and employ a methodology that more precisely assesses the availability, price, and promotion of SSBs to young immigrant children within independently owned grocery stores. A case comparison design was used to explore the differences in the grocery store landscape of SSB marketing by conducting an enhanced Nutrition Environment Measures Survey-SSB (NEMS-SSB) within 30 grocery stores in the Hispanic and Latino enclaves in Southwest Detroit, in the Arab and Chaldean enclaves in North-central Detroit, and in Warren, Hamtramck, and Dearborn, in comparison with 48 grocers in Metro Detroit. Unsweetened, plant-based, and organic toddler and infant beverages, as well as questions about marketing, were added to the original NEMS to capture the promotion tactics used in marketing SSBs. NEMS-SSB scores revealed that, in the immigrant enclaves, there was a significantly higher availability of SSBs in grocery stores (−2.38), and they had lower prices than those in the comparison group (−0.052). Unsweetened, plant-based, and organic beverages were unavailable in 97% of all participating grocery stores across both groups. Signage featuring cartoon characters was the most frequent in-store SSB marketing tactic across both groups. Widespread SSB marketing toward toddlers within the grocery stores in immigrant enclaves could be linked with the higher early childhood obesity prevalence among the immigrant population. Our findings can assist local and national organizations in developing and implementing healthy eating interventions. This study must be repeated in other immigrant enclaves across states to provide comparable results.

## 1. Introduction

Obesity in the United States (U.S.) is the second most noticeable cause of avoidable death for common chronic diseases such as cardiovascular disorders [1,2]. Adult and childhood obesity rates have considerably increased in recent years [1,2]. The childhood obesity prevalence rate in the U.S. nearly tripled between 1980 and 2000 [1,2,3]. Between the years 2017 and 2020, the prevalence of childhood obesity reached 19.7%, and nearly 14.7 million children aged 2–19 years were affected [1,2,3]. In the same period of time, an estimated 12.7% of children younger than five years old, 20.7% of children six to eleven years old, and 22.2% of children twelve to nineteen years old were diagnosed with obesity [1,2,3]. Childhood obesity has historically impacted low-income ethnic minority families [4,5]. Hispanic children (26.2%) and African American children (24.8%) have obesity rates 1.8 times that of non-Hispanic White children (16.6%) [1,3]. Nearly 26% of Latino children are obese at three years of age compared with 16.2% of African American children and 14.8% of White children [1,3,6]. Likewise, 48.8% of Mexican American children have a Body Mass Index (BMI) at the 85th percentile or above, compared with only 29.3% of their peers who live in Mexico [1,3,7]. The same high pattern of obesity also exists among adults from ethnic minorities [1,2,4,5]. The prevalence of obesity is 41.8% among non-Hispanic Black adults and 40% among Hispanic adults, compared with 32.8% among White non-Hispanic adults [1,2,5]. Given that this study was conducted in Metropolitan Detroit, consideration for the Middle Eastern/Arab immigrant population was needed since they comprise a significant number of immigrants within our setting [8,9]. Surveys conducted by the Arab Community Center for Economic and Social Services (ACCESS) also reported a high prevalence of diet-related disorders such as type II diabetes among Arab immigrants, which has an overall prevalence of 15.5% in women and 20.1% in men [8]. However, because of the lack of inclusion within the U.S. Census, there are no separate and precise data regarding Arab immigrants’ health in the national database [9]. Previous research has revealed that ethnic minority individuals who reside in the U.S., such as Hispanic and Arab immigrants, are more susceptible to the risk factors for developing obesity and other chronic diet-related disorders in comparison with the White population [1,2,3,8,9].

Immigrants usually develop patterns of obesity and type II diabetes within one or two generations of moving to the U.S. [10,11,12]. Since genetic changes cannot appear this quickly, environmental factors are the primary determinants for this shift and for shaping nutrition-related behaviors [12,13,14,15]. This is especially true for low-income ethnic minority families who have limited access to healthy food resources, often because of financial, transportation, and linguistic barriers, and who thus rely on convenience and corner stores within close neighborhoods for purchasing food and beverages [16,17,18,19,20,21,22,23]. Grocery stores within low-income neighborhoods typically have lower-quality foods with less variety and are the main source of energy-dense nutrition-poor foods and sugar-sweetened beverages [18,22,24]. In such an obesogenic eating environment, chronic diet-related disorders flourish because of many contributing factors, including the overabundance of unhealthy foods and sugary beverages [22,24,25]. Therefore, improving nutritional environments could be essential in slowing health risk development among health-sensitive populations [12,24,26,27].

The connection between food environments, unhealthy food marketing, and childhood obesity needs to be assessed, and strategies to mitigate environmental contributors should be further developed and implemented [18,23,28,29,30]. Strategies focused on improving the food environment have been found to be effective in improving the consumption of healthy foods and beverages [27,31]. SSBs are the primary source of added sugar leading to weight gain in the American diet and are responsible for half of the sugar consumed by children in the U.S. [1,2,11,32]. According to the American Heart Association, the recommended level of SSB consumption is between 100 and 150 calories per day. However, in 2011–2014, 63% of American youth consumed 143 calories daily from SSBs [33]. The consumption of added sugar should be limited to approximately 10% of an individual’s total daily energy; however, currently, 59% of children who are two to four years old are exceeding this limit of added sugar consumption [34]. Additionally, disparities in SSB consumption can also be seen among Latino/Hispanic and African American groups compared with White children. Latino infants and toddlers have been found to consume more SSBs than their White peers. By age two, 74% of Latino children and 82% of African American children were found to have consumed some SSBs, compared with only 45% of non-Hispanic White children [32,35,36]. SSB marketing impacts children’s diets and eating practices by changing their food preferences and purchase requests [37,38,39]. Furthermore, toddlers have biological preferences for salty and sweet foods [40,41,42]. A lack of cognitive skills also prevents young children from understanding the effects of consuming unhealthy foods [42]. In addition, children’s recognition of food logos increases with age. The children who are exposed to specific logos more commonly prefer selecting those groups of foods and beverages in adulthood [40,43]. It is also hard to change children’s habits of consuming high-sugar foods when they get older [43,44]. These facts make children more vulnerable to SSB marketing within grocery stores, which negatively influences practicing and sustaining healthy eating habits [40,44,45]. Due to the high rates of childhood obesity among Latinos and African Americans, targeted SSB marketing within grocery retail outlets could be an environmental risk factor for that. Such environmental factors negatively contribute to the high rate of SSBs purchase and consumption among Hispanic and non-Hispanic Black children compared with White non-Hispanic children [13,45,46].

Approximately 42,228,200 immigrants live in the U.S. (13% of the population), of which there are 639,500 immigrants in Michigan alone. The Wayne and Macomb Counties in MI host nearly 401,100 immigrants, 63% of the state’s foreign-born population [47,48]. Michigan has the second largest Arab population with much of the population in the immigrant enclaves of Metro Detroit. It is estimated that around 10% of the MI population is of Arab ancestry, the largest Arab population outside the Middle East. Likewise, nearly 7.8% of Metro Detroit’s population consists of Hispanics and Latinos. For many decades now, Southwest Detroit has been known as a Mexican town, one of the main destinations for Hispanic and Latino immigrants. This study assessed the SSBs’ access to Hispanic/Latino and Arab families as a primarily immigrant population of Metro Detroit. This assessment was conducted by evaluating the nutrition environments of the independent grocery stores located in the immigrant enclaves of Wayne and Macomb Counties. Immigrant families who are living in these counties often have a high prevalence of diet-related disorders [47,48]. The adult obesity rate in Wayne County is 35%, which is higher than the state level of 33%. Likewise, Macomb County has an adult obesity rate of 31.9% [47,48]. Residents of these counties also have limited access to healthy eating environments, impacting their young children’s nutritional behaviors and their health outcomes [47,48]. In recent years, there have been many efforts to assess and improve grocery retail environments; however, comparatively little is known about which aspects of the grocery environment influence immigrant parents’ purchasing of SSBs for their toddlers. There is also a gap in the literature measuring the marketing aspects of SSBs, such as the availability, price, and promotion of SSBs to young children within independent grocery stores [30,49].

Developing and pilot testing a tool that can correctly and constantly assess the availability, price, and promotion of SSBs to two- to five-year-old children within grocery stores was the main goal of the current study. Employing an enhanced version of the Nutrition Environment Measures Survey in Stores (NEMS-S) assists in measuring SSB accessibility and affordability. This study aimed to address these knowledge gaps by (1) expanding an enhanced measure of the NEMS-S or NEMS-SSB to include an assessment of the availability, price, and promotion of SSBs to immigrant families of young children in the grocery retail environment and (2) distinguishing differences in the landscape of SSB marketing across retail grocery settings in immigrant enclaves when compared with a socio-culturally different area. This study adds to the literature on early childhood obesity and highlights the connection between the impact of SSB marketing within retail grocery settings and the nutritional behaviors of young children.

## 2. Materials and Methods

A cross-sectional case-comparison approach was used to explore NEMS-SSB scores, which were obtained through assessing the nutritional environments of the participating grocery stores by employing the NEMS-SSB tool [50,51]. Availability, price, and promotion of the SSBs within 78 independently owned grocery stores, including 30 grocery stores in Hispanic and Latino enclaves in Southwest Detroit and Arab and Chaldean enclaves in north-central Detroit, Warren, Hamtramck, and Dearborn, and 48 grocery stores in Metro Detroit as a comparison group, were assessed. The comparison grocery stores serve a part of Detroit’s population that is less diverse than the population of the immigrant enclaves. These independently owned grocery stores were located in the Metro Detroit and the surrounding area <2 miles (<3.2 km) of the city limits identified using the 2021 food store database from the Michigan Department of Agriculture and Rural Development (MDARD) [52]. Google searches verified the store addresses. If the stores were found to be closed, they were contacted by phone calls or by an in-person drive-by to verify their existence. In total, 84 independently owned grocery stores were identified. Of these, NEMS-SSB was conducted in 78, *n* = 30 in immigrant enclaves and *n* = 48 in comparison group, and quantitative data were collected by trained research assistants through April–May 2022. The stores that were not surveyed by NEMS-SSB were permanently closed (*n* = 6). No significant differences were detected between closed and surveyed grocery stores with respect to neighborhood, location, and store type. The NEMS-SSB was enhanced from the original NEMS-S, a previously nationally validated survey developed by Glanz et al. [50,51]. The original NEMS-S did not measure the availability, price, and promotion of some specific types of SSBs for toddlers and young children in grocery stores. The toddler and infant drink categories were modified to include unsweetened, organic, and regular powder formula, ready-to-feed formula, soy formula, toddler milk, and pediatric drinks such as Pediasure or Pedialyte. The milk and dairy category was also modified to include unsweetened, plant-based, organic, and regular plain milk, flavored milk (chocolate, strawberry), kids’ yogurt drinks, and squeezable yogurts. It is necessary to mention that only a few studies discussed that consuming organic products might contribute to reduced obesity risk. However, there is still a lack of solid evidence for this and a need to conduct more extensive research for categorizing organic products as healthier items than regular ones [53,54,55,56,57]. In this study, the reason for adding organic beverages to the original NEMS-S was only to assess the availability and affordability of these products within the targeted stores and measure the access of the target immigrant families to these types of beverages. Additionally, to fully assess SSB marketing, two marketing questions were added at the end of each category to capture the placement and marketing tactics used within grocery stores to promote SSBs to young children, including whether there was any flavored milk on the endcaps of the dairy section. The NEMS-SSB scoring was calculated based on the original NEMS-S scoring protocol [50,58]. Author developed a scoresheet (Table 1). For each store, a total NEMS-SSB score was obtained from adding two sub-scores of SSB availability and affordability. Availability was assessed by counting rows of added beverages in two categories of the milk and dairy, and infant and toddler beverages. Affordability was assessed by comparing the price of any available unsweetened, plant-based, or organic options to the price of the regular beverages or SSBs over-added items in each category mentioned above. These scores were then used to ascertain the SSB marketing level at the store. Based on the developed NEMS-SSB scoresheet, the SSBs’ availability score ranged between −4 and 4. The SSBs’ price score ranged from −2 to 6, and the total NEMS-SSB score ranged between −6 and 10. The grocery stores that provided unsweetened, plant-based, or organic formula received two positive points, and, for regular formula, one positive point, respectively. Having pediatric drinks, toddler milk, kids’ yogurt drinks, and flavored milk of any type caused stores to receive one minus point. Likewise, the grocery stores that provided alternative plant-based milks received positive point. Therefore, minus values for availability scores indicate a higher number of rows for SSB products or higher availability of SSBs in the store. Price scores were assigned by giving 1–2 points to the price of the unsweetened, plant-based, or organic formula and milk that were available at a lower or same price, respectively, than regular ones. Negative values were assigned to the price score if the price of unsweetened, plant-based, and organic options was greater than the price of the regular option in the same group. For price score, if the SSBs were cheaper than other available comparable options, then smaller negative values were assigned to the price NEMS score. The Wayne State University Institutional Review Board (IRB) approved this study (065117B3X). Building on work concerning the community’s access to healthy foods and beverages, we developed an enhanced NEMS tool with an SSB score. To our knowledge, no other studies have evaluated the community nutrition environment using the NEMS-SSB. Additionally, in this study, by connecting the consumer nutrition environment via the NEMS-SSB scores of the grocery stores to the racial makeup or zip code of the neighborhoods, differences in the level of the SSB marketing to young immigrant children within grocery stores across socio-culturally different communities can be observed.

## 3. Statistical Analysis

In the analysis of the quantitative data, the NEMS-SSB mean scores for availability, price, and total points of the grocery stores in the immigrant enclaves of Metro Detroit were compared with the mean scores of grocery stores in the comparison groups. Descriptive statistics and frequencies were generated for the NEMS-SSB scores for both groups. Mean scores were compared using Analysis of Variance (ANOVA). Descriptive and frequency analyses were used for observing the results of the marketing questions to recognize any potential structural racism in accessing unsweetened beverages within the grocers located in the immigrant enclaves. The ANOVA examined the differences in the availability and price of the SSBs, unsweetened beverages, and healthier items for toddlers and infants between the two groups [59]. In this study, the dependent variable was the availability and price of SSBs versus the availability and price of organic or unsweetened beverages. The independent variable was the grocery stores’ groups, with a comparison group of grocers in Metro Detroit designated as one group and the immigrant enclaves of Warren, Hamtramck, Dearborn, and Detroit as another group. Following mean comparisons, Levene’s statistic was used to verify the homogeneity of variances among the dependent variables for the ANOVA. The obtained eta-squared values demonstrated the effect size. The eta-squared values provided additional information on the comparisons of the availability and affordability of the SSBs and unsweetened beverages for toddlers and infants. To check if variables follow a normal distribution, the Shapiro–Wilk test was run. It was significant, but ANOVA was robust despite non-normality, and the Type-I error rate remained constant and did not alter by the violation of normality. Violations of the normality of residuals assumption are not problematic for hypothesis testing [60,61,62,63]. The statistical software package SPSS version 28.0.1.0 (142) (IBM Corporation, Armonk, NY, USA, 2022) was used for all analyses.

## 4. Results

Table 2 and Table 3 highlight results based on the analysis of grocery stores’ NEMS-SSB scores in immigrant enclaves of Metro Detroit and the comparison group, respectively. The results of the descriptive and frequency analysis in Table 2 demonstrate that SSBs are more available (higher number of rows) to consumers within the immigrant enclaves’ grocery stores (M = −1.86) when compared with the comparison grocery stores (M = −0.06) (Table 2). Likewise, Levene’s test verified the homogeneity of variances among SSB availability in both groups (Levene test = 2.50, F (1,75) = 17.69 *p* < 0.001) and eta-squared results also displayed a large effect size with a point estimate of (η^2^ = 0.19, 95% CI [0.055, 0.338]). In confirmation of the observed findings, one-way ANOVA results also indicate that SSB availability is significantly higher within the immigrant enclaves’ grocery stores than those within the comparison group (Table 3). In confirmation of the observed significant differences, the Levene statistic showed homogeneity of variances between the two groups. The mean score for the NEMS-SSB price in the immigrant enclaves’ group (M = −0.46) has a smaller negative value than the mean NEMS-SSB score in the comparison group (M = −0.52), which means that the mean price of SSBs is cheaper in the immigrant enclave group than the mean price of SSBs in the comparison group (Table 2). However, ANOVA results indicate that this difference is not statistically significant between the two groups (Levene test = 0.173, F (1,75) = 0.72 *p* < 0.789) and eta-squared also showed a small effect size with a point estimate of (η^2^ = 0.001, 95% CI [0, 0.54]) (Table 3). The total mean NEMS-SSB score, including availability and price, was higher in immigrant enclaves’ grocery stores (M = −2.38) versus the total mean NEMS-SSB score in comparison grocers (M = −0.052) (Table 2). The ANOVA results also show that the total NEMS-SSB score had a significant difference between the two groups. Levene statistics showed homogeneity of variances. (Levene test = 0.261, F (1,75) = 11.08 *p* < 0.001) and the effect size was also found to be large (η^2^ = 0.129, 95% CI [0.021, 0.0271]), which confirmed a significant difference in total NEMS-SSB scores between the two groups (Table 3).

### 4.1. Milk and Dairy

The descriptive analysis showed that the availability of soy milk (M = 1.57), other plant-based milk (M = 2.39), and flavored milk (M = 2.14) was lower in immigrant enclaves than in the comparison group (soy milk (M = 6.53), plant-based milk (M = 7.20), and flavored milk (M = 3.57)) (Table 4). There was homogeneity of variances among the variables, such as soy milk and other plant-based milk (Levene test = 2.95, F (1,73) = 2.61 *p* < 0.111), (Levene test = 3.39, F (1,72) = 2.30 *p* < 0.134) (Table 4). Although the availability of soy and other plant-based milk as healthier options was higher in the comparison grocery stores, based on the ANOVA findings, this difference was not statistically significant. (Table 4). Similarly, the low availability of flavored milk in the immigrant enclaves’ group (M = 2.14) compared with the comparison group (M = 3.57) was not statistically significant. Eta-squared results also show a small effect size for each milk group, which confirms the insignificance of these differences in the findings (Table 4). As expected, within the immigrant enclaves’ group, the mean availability of soy milk (M = 1.57) was lower than the mean availability of flavored milk (M = 2.14); however, this difference was also not statistically significant (Table 4). The mean availability of kids’ squeeze yogurt (M = 3.25) and yogurt smoothies (M = 2.29) in the immigrant enclaves’ group was slightly lower compared with the mean availability of the squeeze yogurt (M = 3.57) and yogurt smoothies (M = 2.86) in the comparison group, and this difference was also not statistically significant (Table 5). The mean price of soy milk (M = 2.69) and other plant-based milks (M = 2.22) was lower in the immigrant enclaves’ group than the mean price of soy milk (M = 3.61) and other plant-based milks (M = 3.1) in the comparison group. Statistically speaking, observed differences among the prices of different milk and dairy products across the grocery stores in both groups were not significant. Lower prices for the plant-based and soy milks in the immigrant enclaves’ group might be due to a low availability of such products in that group. In accordance with the mean, variances had homogeneity for price except for the price of soy milk with the significant Levene test (19.551, F (1,72) = 4.29 *p* < 0.001) (Table 5), and the results of the ANOVA show an insignificance in price differences (Table 5).

The descriptive analysis showed that, within the immigrant enclaves’ grocery stores, the mean price of flavored milk (M = 2.95) was slightly higher than the mean price of soy milk (M = 2.69) and higher than the mean price of the plant-based milks (M = 2.22), which indicates that the soy milk within the immigrant enclaves’ grocery stores and between the two groups had a lower price. However, based on the results of the ANOVA, this difference is not statistically meaningful and could be due to the low availability of the soy and plant-based milk within the immigrant enclave grocery stores (Table 6). The mean price of the squeeze yogurt (M = 2.07) and yogurt smoothies (M = 2.27) in the immigrant enclaves’ group was slightly lower compared with the mean price of the squeeze yogurt (M = 2.10) and yogurt smoothies (M = 2.34) in the comparison grocery stores. Based on the mean and the results of the ANOVA, the price of the squeeze and smoothie yogurts had no significant differences across the two groups. The effect size was also found to be small (η^2^ = 0.000, 95% CI [0.000, 0.0.21]), demonstrating that squeeze and smoothie yogurt had a similar price in both store groups. In conformity with the mean, the variance was not homogeneous for the price of the yogurt smoothie with the Levene test (7.71, F (1,73) = 0.17 *p* < 0.897) (Table 6).

### 4.2. Toddler and Infant Milk

The results of descriptive analysis show that the availability of the regular formula was higher in the immigrant enclaves group (M = 4.06), with a higher price (M = 16.78) than the comparison grocery stores, which had an availability of M = 3.93 and price of M = 15.20; however, this difference was not statistically significant. Pediatric nutritional drinks were less available in the immigrant group (M = 4.39) and were cheaper (M = 2.93) relative to the comparison group (availability M = 6.24; price M = 4.53). These differences were not statistically significant. Likewise, there was no significant difference between the availability of toddler milk in the immigrant enclaves group (M = 3.33) and the comparison group (M = 3.28). Levene statistics showed homogeneity of variances except for the availability of organic toddler milk (Levene test = 20.42, F(1,45) = 3.715 *p* < 0.001) and for the price of organic toddler milk (Levene test = 27.53, F(1,45) = 4.213 *p* < 0.001) (Table 7). The availability of organic formula, toddler milk, and pediatric nutritional drinks was zero or at most one row of products in both groups. In the immigrant enclaves group, only 1 grocery store out of 30 offered organic formula, and in the comparison group, only 3 out of 48 grocery stores offered organic formula; therefore, there was nothing to compare. Within the immigrant enclaves’ grocery stores, the mean price of the pediatric nutritional drinks was M = 2.93, and the mean price of toddler milk was M = 14.5, both of which were cheaper than the mean price of the formula (M = 16.78). However, as explained above, the lower mean price could be due to the low availability of the products, as well as the low availability of organic infant and organic toddler milks (Levene test = 27.538, F(1,45) = 4.213 *p* < 0.001); thus, there was no possibility of comparing the price of the toddler and infant products between and among the groups given the limited items across all stores (Table 8).

### 4.3. Marketing Questions

The descriptive analysis showed that flavored milk was placed on the endcaps of the dairy section in 33% (10 out of 30) of the immigrant stores versus only 6% (3 out of 48) of comparison stores. In 56% of the immigrant enclaves’ stores (17 out of 30), there were kids’ yogurt drinks on the endcaps of the dairy section versus in 91% in the comparison group (44 out of 48). In almost 97% of the grocery stores in both groups, there were marketing materials/signage featuring cartoon characters near the milk/dairy/yogurt section. Likewise, for the toddler and infant drinks, in 97% of the grocery stores in both groups, there were toddler or infant drinks on the endcap aisles, along with marketing materials/signage featuring cartoon characters near the toddler and infant drink section (Table 9 and Table 10).

## 5. Discussion

This study aimed to enhance the NEMS-S to evaluate retail-based SSB marketing to young children within the independently owned grocery stores in the Metro Detroit, Dearborn, Hamtramck, and Warren, MI. The study used the NEMS-SSB to assess the availability and price of SSBs and the promotion tactics used to market them within grocery stores. NEMS-SSB scores were used to assess SSB marketing for infants and toddlers within the independently owned grocery stores in the immigrant enclaves group and compare this with the comparison group in Metro Detroit. NEMS-SSB scores also depicted how other retail variables, such as price, and in-store marketing strategies, such as the placement of SSBs, impact the stores’ SSB scores. In this study, the results indicate that statistically significant differences in SSB availability were present, with parents and caregivers reporting a wider variety of SSBs in immigrant enclave communities. The environmental scanning recorded a high abundance of SSBs within grocery stores in the immigrant enclaves compared with the comparison group in Metro Detroit, where the majority of resident families do not identify as immigrants [64,65].

At this level of significance, the results also verify that the high density of SSBs within the immigrant enclaves’ grocery stores could be associated with a high SSB consumption among young children and adolescents of color [66]. Likewise, increases in in-store SSB marketing can act as a potential environmental contributor for the high rate of early childhood obesity among the ethnic minority population, such as Hispanic Latino, Arabs, and African American, who comprise the majority of residents in immigrant enclaves in the Wayne and Macomb Counties in this study [35,36,67]. Similar to the results of previous research, it was found that, within predominantly non-White neighborhoods, either there was no access to supermarkets at all, or the only available supermarkets were non-healthy [44,45]. Lower access to healthy supermarkets is associated with a lower consumption of healthy foods and beverages [68,69]. Notably, the findings of this study demonstrate that the availability of organic, unsweetened infant and toddler beverages and plant-based milk and dairy products was slim to none in both groups of grocery stores, which is an indicator of healthy beverage insecurity in these areas. Healthy food and beverage insecurity can also aggravate pre-existing health conditions, such as obesity and type II diabetes, and could be another potential reason for why immigrant families suffer a higher prevalence of obesity compared with White populations [70,71]. These findings are consistent with the findings of the previous studies that state that a greater availability of energy-dense nutrition-poor foods and sugary beverages within grocery stores results in a higher prevalence of severe diet-related metabolic disorders among consumers [70,71].

In accordance with the mean price score of SSB products, SSBs were more affordable and had a lower price in the immigrant enclaves’ group relative to the comparison group in Metro Detroit. However, this difference was statistically insignificant. The price of organic and unsweetened beverages (if any were present) was significantly higher than the price of regular beverages and milk across all grocery stores in both groups. Immigrant enclaves are host to high number of low-income families who cannot afford to purchase expensive unsweetened or organic beverages and are more heavily targeted by SSB promotion and discounts [37,70,71]. Although NEMS-SSB price scores and influence on consumer purchases were not assessed in this study, as identified in previous research, pricing interventions strongly impact food and beverage consumption. Thus, lower prices for SSBs may be another reason for the overall high consumption of them, indicating unhealthy nutritional behaviors [27,51]. Higher prices for SSBs may have a protective influence on shaping healthy nutritional behavior [27,51]. Further analyses should explore the potential effects of the discounting, low pricing, and promotion of unsweetened beverages for toddlers and infants on health-related outcomes such as obesity.

The current study provides further insight into overall SSB marketing to young children using the NEMS-SSB tool and its findings show that, within grocery stores located in the immigrant enclaves, SSBs are highly marketed to young children, indicating that, overall, fewer unsweetened beverages were available, and these were not promoted. The study revealed that SSB placement on the endcaps of the dairy, toddler, and infant drink sections, alongside the use of signage featuring cartoon characters, is the dominant approach in SSB marketing to young children within the participating grocery stores. As a result, practicing healthy eating behaviors may be more difficult for families who are consumers of the non-supportive grocery store environments [72,73]. Moreover, several other studies explained that those children with minority and ethnic backgrounds are more greatly affected by such targeted SSB marketing strategies [28,30,74], and this is associated with a higher prevalence of obesity among them in comparison to White children [3,4,28].

Additionally, access to healthier grocery food environments is associated with access to high-quality and healthy foods and beverages and can serve as a potential contributor to healthy food security within communities of color in Metro Detroit [16]. Other research studies have indicated that food stores are health promotion agents and can aid in improving the healthy eating behavior of families [16,70,74,75,76]. The comparison of the NEMS-SSB scores revealed the negative influence of SSB marketing on children’s improving or deteriorating nutritional behaviors. Although SSB marketing and consumption contribute to childhood obesity, specifically among immigrant young children [28,33,73], to our knowledge, no other study has evaluated SSBs’ availability, affordability, and promotion to infants and toddlers within independently owned grocery stores using the NEMS-SSB until now. By enhancing the original NEMS-S to become the NEMS-SSB, this study presents a first assessment of the grocery store environment on SSB marketing to young children. The results of conducting this new methodology, through connecting the store level of SSB marketing and the location level of the grocery stores, shows implications for restricting in-store SSB marketing to serve as a protective factor for reducing early childhood obesity prevalence among immigrant populations. This assessment method can be replicated in other immigrant enclaves across states to provide comparable results and can be used to inform local and national organizations to make efforts to improve community food environments and increase the availability and marketing of unsweetened and healthy beverages for young children. Furthermore, the results of this study could be applied to grocery stores that accept WIC (Supplemental Nutrition program for Women, Infants and Children) and SNAP (Supplemental Nutrition Assistance Program) through the regulation of SSB marketing within store environments; for example, removing SSBs from children’s eye level in grocery stores and encouraging grocery store owners/managers to develop healthy checkouts that include substituting SSBs in checkouts with unsweetened beverages [77,78].

The secondary goal of this study was to use the NEMS-SSB as a means of connecting the consumer nutrition environment to the sociodemographic characteristics of neighborhoods, such as race. As mentioned earlier, race is a significant determinant for access to healthy foods [75,76] and, historically, some communities, such as immigrants, had lower access to healthy food sources [75,76]. The findings of this study outline the results for SSB abundance in the independent grocery stores in the immigrant enclaves in Metro Detroit, which is home to primarily Black/African American, Hispanic/Latino, and Arab immigrant families, which makes them more susceptible to diet-related disorders [2,3,6,8,9]. The findings of this study can connect SSB scores to neighborhood socio-economic characteristics to examine determinants such as income and determining inequalities in access to healthy foods and beverages.

The present study has many advantages, including introducing the NEMS-SSB tool to evaluate grocery store environments and providing a comprehensive picture of SSB marketing within grocery stores. However, there are also limitations. First, the NEMS-SSB has not been used in any other studies, so its applicability requires further research. Second, inter-rater reliability tests on the NEMS-SSB were only undertaken in one store and the other participating stores were evaluated only once. Likewise, grocery store environments were assessed individually or by a team of two student assistants to save time. However, the data collectors in this study were certified NEMS-S raters or had been trained by the certified NEMS-S raters. Additionally, the target population of this study was Arab/Chaldean and Hispanic/Latino groups; therefore, the generalizability of the results is limited, and research should be conducted in other immigrant communities to make the results more generalizable. Finally, establishing healthy nutritional behavior is complicated and multivariate and is highly influenced by several social determinants; thus, acquired data in this study only provide an outline on which to define the potential impact of nutritional environments on forming nutritional behavior and practicing healthy eating habits. Future examinations will link authenticated measures of children’s SSB consumption to understand the role of the SSB marketing/healthy beverage marketing in the establishment and maintenance of healthy nutritional behavior among young immigrant children.

This paper is important in relation to the other current efforts being made to bridge the gaps in healthy food access within Metro Detroit. We have already collected preliminary NEMS-SSB data for initiatives with the aim of developing and implementing food-retail-based healthy interventions. One such ongoing effort is the Great Grocers Project (GGP), which has a focus on in-store healthy food marketing [64]. The GGP is using data derived from this study to develop and implement culturally tailored healthy food marketing within the independently owned grocery stores of the Metro [64]. Data such as ours will assist in the GGP by recognizing grocery stores in which there is a high SSB marketing level. The Detroit Food Policy Council is currently collaborating with the GGP to plan and implement healthy food marketing within the grocery stores in low-income and underserved neighborhoods of Metro Detroit. Lastly, although larger chain grocers are often the best source for the purchase of healthy foods and beverages, they are not always the first source for immigrant families. Therefore, improving the eating environment of the independently owned grocery stores is a challenge in meeting the basic requirements for promoting healthy outcomes among consumers.

## 6. Conclusions

By developing the NEMS-SSB, we offer the first reliable methodology for assessing SSB marketing within the nutritional environment of independently owned grocery stores as evidence of the inequity in the availability and promotion of unsweetened beverages for toddlers and infants who are living in immigrant enclaves compared with other neighborhoods in the Metro Detroit area. Independently owned grocery stores within the immigrant enclaves of Metro Detroit—Dearborn, Hamtramck, and Warren—were found to be associated with a greater availability and promotion of SSBs and the potential negative impact of the obesogenic environment on the nutritional pattern. Previous food access studies have been limited by having no separate category for toddler and infant beverages to assess the marketing aspects. The NEMS-SSB is a novel assessment for recognizing potential imbalances in healthy food and beverage access for minority families and provides a meaningful contribution to the literature. Such a contribution advances understanding, which leads us to the development and implementation of an appropriate healthy nutrition intervention to address early childhood obesity among underserved populations. Further investigation into equitable, healthy food and unsweetened beverage access as potential protective factors for early childhood obesity is needed. Researchers in other immigrant communities may employ this enhanced tool and replicate the evaluation to gain comparable data about SSB marketing for young children in their local grocery stores. Then, the generalizability of this enhanced tool will be determined. Such work expands the understanding of unsweetened beverage accessibility and design and the implementation of interventions in creating healthy retail settings in order to address inequity in healthier food and beverage access for low-income immigrant communities.

## Figures and Tables

**Table 1:** SSB assessment scoring sheet.

Item	Availability	Price
Formula	Unsweetened/plant-based/organic = 2 pts	Lower for unsweetened/plant based/organic = 2 pts
	Regular = 1 pt	Same for both = 1 pt Higher for unsweetened/plant-based/organic = −1 pt
Toddler milk	Any type = −1 pt	
Pediatric drinks	Any type = −1 pt	
Milk	Alternative (e.g., soy, almond) = 1 pt	Lower for alternative = 2 pts
	Flavored milk = −1 pt	Same for both = 1 pt
		Higher for alternative = −1
Kids’ yogurt drinks	Any type = −1 pt	
	Availability range (−4 to 4)	Price range (−2 to 6)
Total NEMS-SSB score range	(−6 to 10)	

**Table 2:** Results of the NEMS-SSB scoring means.

Grocery Store Group	SSB Availability (Number of Rows) (Range −4 to −4)	SSB Price (Range −2 to 6)	Total (Range −6 to 10)
Immigrant Enclaves (*n* = 30)	−1.86	−0.46	−2.38
Comparison Stores (*n* = 48)	−0.06	−0.52	−0.052

**Table 3:** Results of the NEMS-SSB scoring ANOVA Sig.

Grocery Store Group	Sum of Squares	df	Mean Square	F	Sig.
Between Groups	58.544	1	58.544	17.686	<0.001
Within Groups	248.261	75	3.310		
Between Groups	0.063	1	0.063	0.072	0.789
Within Groups	65.158	75	0.869		
Between Groups	62.440	1	62.440	11.76	<0.001
Within Groups	422.807	75	5.637		

**Table 4:** Results of the milk and dairy availability scoring ANOVA Sig.

Number of Row	Sum of Squares	Df	Mean Square	F	Sig.
Other milk alternative				2.296	0.134
Between Groups	401.488	1	401.488		
Within Groups	12,589.918	72	174.860		
Soy milk				2.610	0.111
Between Groups	431.761	1	431.761		
Within Groups	12,076.559	73	165.432		
Flavored milk (chocolate)				1.003	0.320
Between Groups	35.962	1	35.962		
Within Groups	2616.918	73	35.848		
Flavored milk (strawberry)				1.658	0.202
Between Groups	62.113	1	62.113		
Within Groups	2735.033	73	37.466		

**Table 5:** Results of the kids’ and yogurt drinks availability scoring ANOVA Sig.

Number of Row	Sum of Squares	Df	Mean Square	F	Sig.
Kids’ squeeze yogurt				0.128	0.722
Between Groups	1.847	1	1.847		
Within Groups	1056.739	73	14.476		
Kids’ yogurt smoothies				0.604	0.439
Between Groups	5.864	1	5.864		
Within Groups	708.203	73	9.701		

**Table 6:** Results of the milk and dairy price scoring ANOVA Sig.

Number of Row	Sum of Squares	Df	Mean Square	F	Sig.
Flavored milks				1.345	0.250
Between Groups	2.478	1	2.478		
Within Groups	134.482	73	1.842		
Soy milk				4.299	0.042
Between Groups	14.763	1	14.763		
Within Groups	247.281	72	3.434		
Other milk alternative				4.550	0.036
Between Groups	16.620	1	16.620		
Within Groups	266.670	73	3.653		
Kids’ squeeze yogurt				0.005	0.949
Between Groups	0.013	1	0.013		
Within Groups	203.817	72	2.831		
Kids’ drink yogurts/smoothies				0.017	0.897
Between Groups	0.070	1	0.070		
Within Groups	304.870	73	4.176		

**Table 7:** Results of the toddler and infant milk availability scoring ANOVA Sig.

Number of Row	Sum of Squares	Df	Mean Square	F	Sig.
Formula				0.007	0.935
Between Groups	0.172	1	0.172		
Within Groups	114.807	45	25.440		
Formula (organic)				1.820	0.184
Between Groups	1.365	1	1.365		
Within Groups	33.741	45	0.750		
Toddler milk				0.002	0.963
Between Groups	0.037	1	0.037		
Within Groups	767.793	45	17.062		
Toddler milk (organic)				3.715	0.060
Between Groups	1.902	1	1.902		
Within Groups	23.034	45	0.512		
Pediatric nutritional drinks				1.072	0.306
Between Groups	38.114	1	38.114		
Within Groups	1599.588	45	35.546		
Pediatric nutritional drinks (organic)				0.619	0.436
Between Groups	0.65	1	0.065		
Within Groups	4.743	45	0.105		

**Table 8:** Results of the toddler and infant milk price scoring ANOVA Sig.

Number of Row	Sum of Squares	Df	Mean Square	F	Sig.
Formula				0.177	0.676
Between Groups	27.766	1	27.766		
Within Groups	7057.764	45	156.839		
Formula (organic)				0.265	0.609
Between Groups	50.463	1	50.463		
Within Groups	8577.129	45	190.603		
Toddler milk				1.930	0.172
Between Groups	240.647	1	240.647		
Within Groups	5609.834	45	124.663		
Toddler milk (organic)				4.213	0.046
Between Groups	598.869	1	598.869		
Within Groups	6396.639	45	142.148		
Pediatric nutritional drinks				1.741	0.194
Between Groups	14.559	1	14.559		
Within Groups	376.234	45	8.361		
Pediatric nutritional drinks (organic)				0.616	0.437
Between Groups	0.954	1	0.954		
Within Groups	69.759	45	1.550		

**Table 9:** Milk and dairy marketing strategies.

Marketing Strategy	*n*	Mean
Is there any flavored milk on the endcaps of the dairy section?		
Immigrant Enclave Groups	10	0.33
Comparison Groups	47	0.6
Are there any kids’ yogurts/drinks on the endcaps of the dairy section?		
Immigrant Enclave Groups	17	0.56
Comparison Groups	44	0.91
Are there any marketing materials/signage featuring cartoon characters near the milk/dairy/yogurt section?		
Immigrant Enclave Groups	30	0.97
Comparison Groups	47	0.97

**Table 10:** Toddler and infant drinks marketing strategies.

Marketing Strategy	*n*	Mean
Is there any flavored milk on the endcaps of the dairy section?		
Immigrant Enclave Groups	30	0.41
Comparison Groups	48	0.06
Are there any kids’ yogurts/drinks on the endcaps of the dairy section?		
Immigrant Enclave Groups	30	0.41
Comparison Groups	48	0.08

## Data Availability

The data presented in this study are available by request from the corresponding author. NEMS raw scores of stores are not shared publicly to protect the independent grocers in Detroit. Collective ratings of each store with a listing of each category meeting the requirements of the Great Grocer Project can be found on the Detroit Food Policy Council/Detroit Grocery Coalition website: https://www.detroitfoodpc.org/committees/#dgc (accessed on 15 May 2023).
